# Supplementary figures and images for: Structural basis for carbohydrate recognition by the Gal/GalNAc lectin of Entamoeba histolytica involved in host cell adhesion
Source: PLoS Pathog. 2026 Feb 24;22(2):e1013948. doi: 10.1371/journal.ppat.1013948 (PMC12948311; doi:10.1371/journal.ppat.1013948)

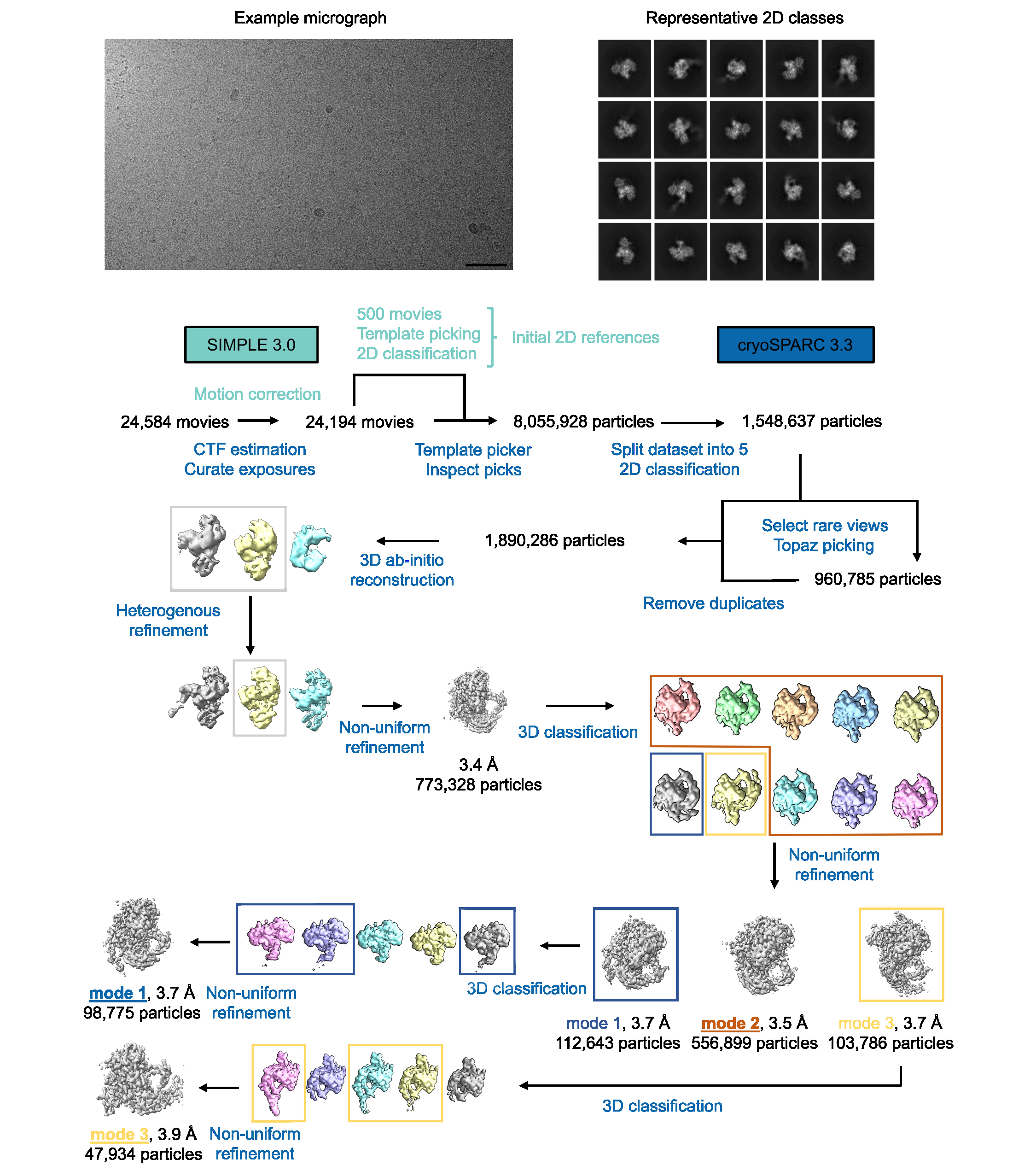

Supplement: S1 Fig — The scale bar corresponds to 50 nm. (TIF) [file ppat.1013948.s001.tif]

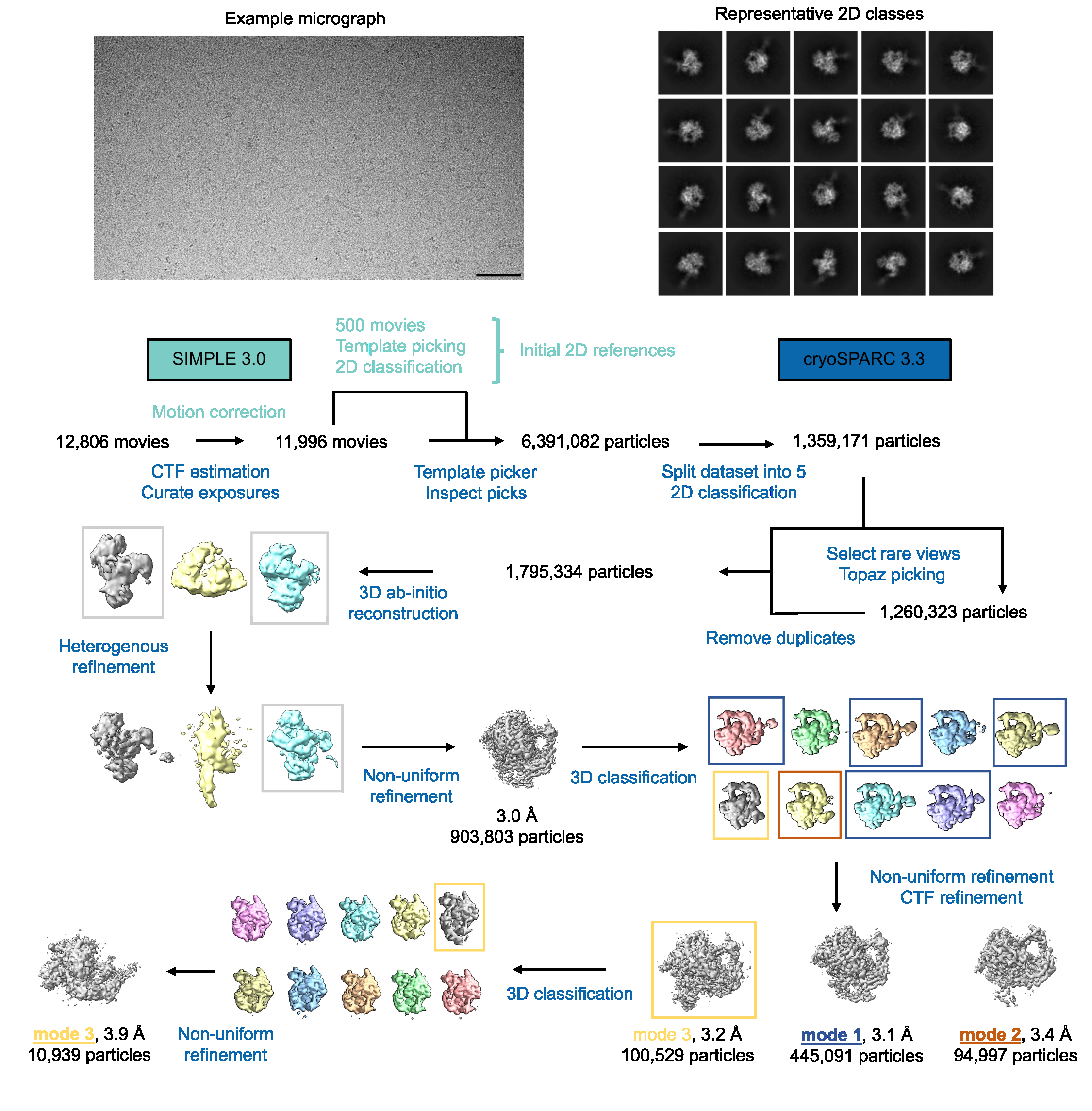

Supplement: S2 Fig — The scale bar corresponds to 50 nm. (TIF) [file ppat.1013948.s002.tif]

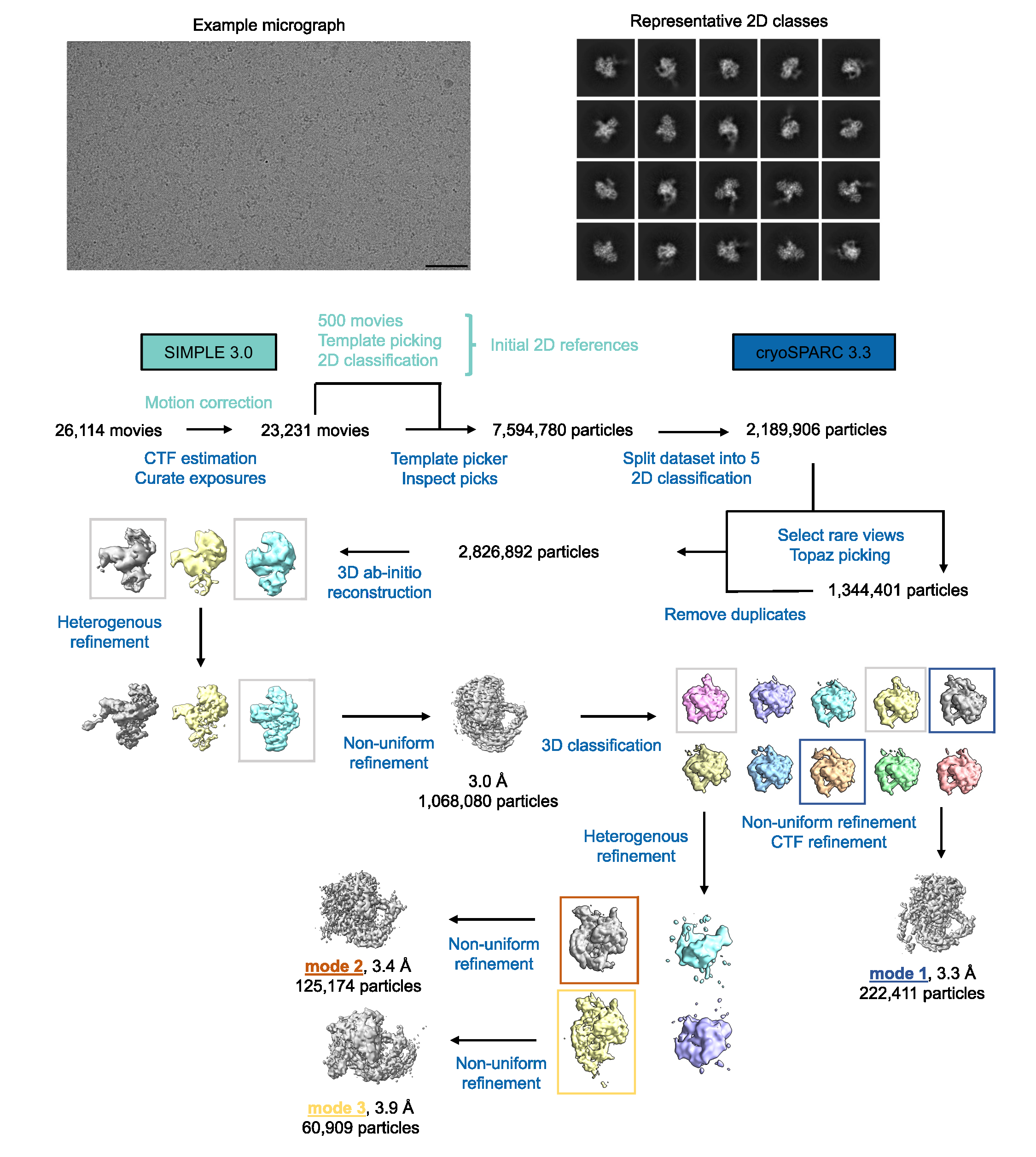

Supplement: S3 Fig — The scale bar corresponds to 50 nm. (TIF) [file ppat.1013948.s003.tif]

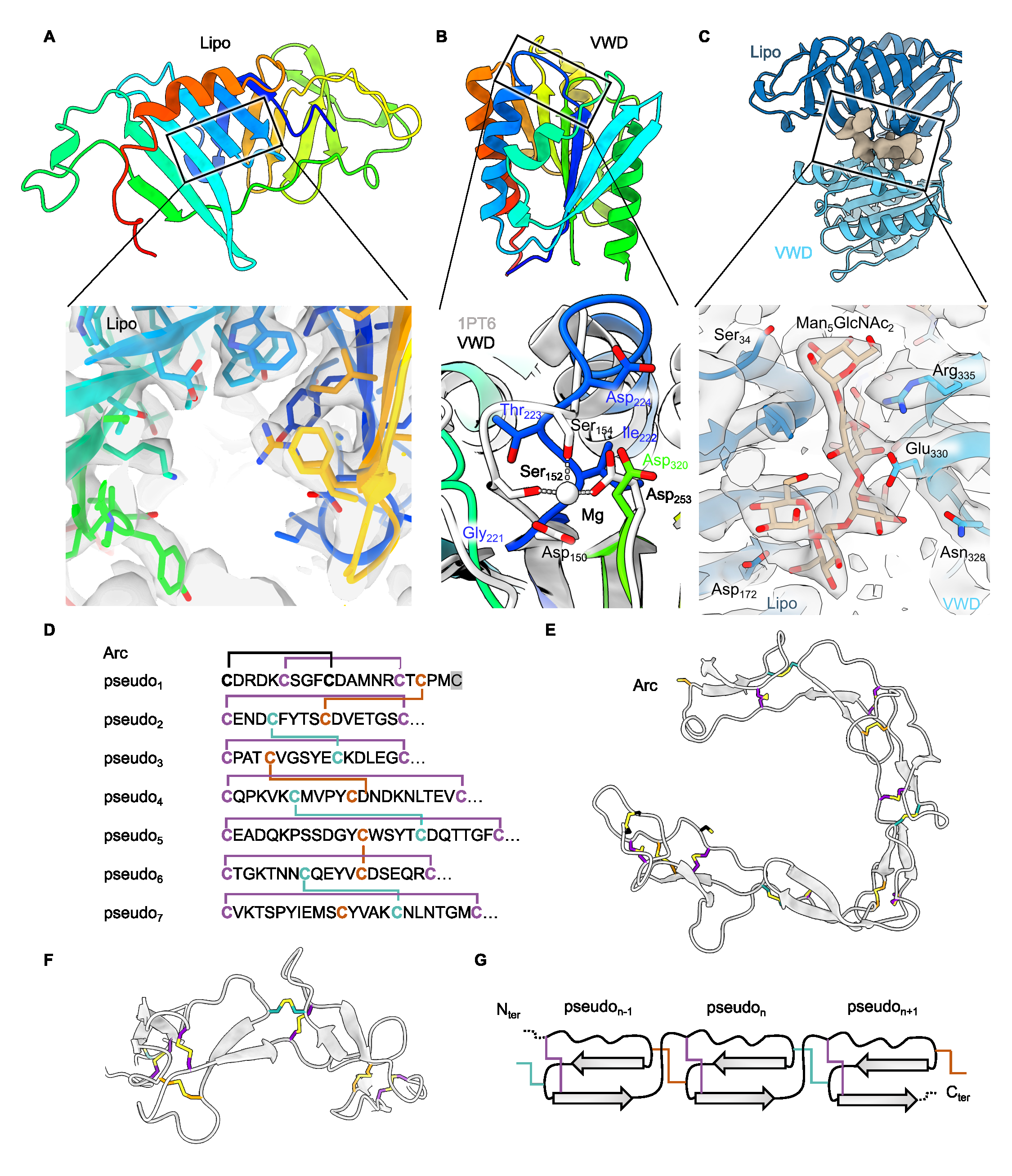

Supplement: S4 Fig — (A) Rainbow representation of the lipoprotein-like domain of HgL (Lipo). The rectangle highlights the site which binds lipid-like molecules in homologous structures. Below is a close-up showing the electron density in the cavity and at the centre of the lipoprotein-like domain, revealing the absence of density due to a ligand. (B) Rainbow representation of the von Willebrand-like domain of HgL (VWD). The location of a potential MIDAS is delimited by a rectangle. A close-up showing the structural alignment of the VWD (rainbow) and the MIDAS of α1-β1 integrin (PDB: 1PT6, grey) is displayed below. (C) Cartoon representation of the Lipoprotein-like domain (Lipo, dark blue) and the von Willebrand-like domain (VWD, light blue), overlaid with the electron density of the N-glycan linked to Asn390 (brown) stabilising the interface between both domains. Close up on the Man5GlcNAc2 glycan at the interface between the lipoprotein-like domain and the von Willebrand-like domain. (D) Sequence of each pseudo-domain, with cysteines coloured. Bridges between cysteines represent disulphide bonds. (E) Cartoon representation of the arc of pseudo-domains with disulphide bonds coloured as (A). (F) A close-up on a portion of the arc of pseudo-domains showing the interconnection between adjacent pseudo-domains. (G) Schematic representation of the topology adopted by the pseudo-domains. (TIF) [file ppat.1013948.s004.tif]

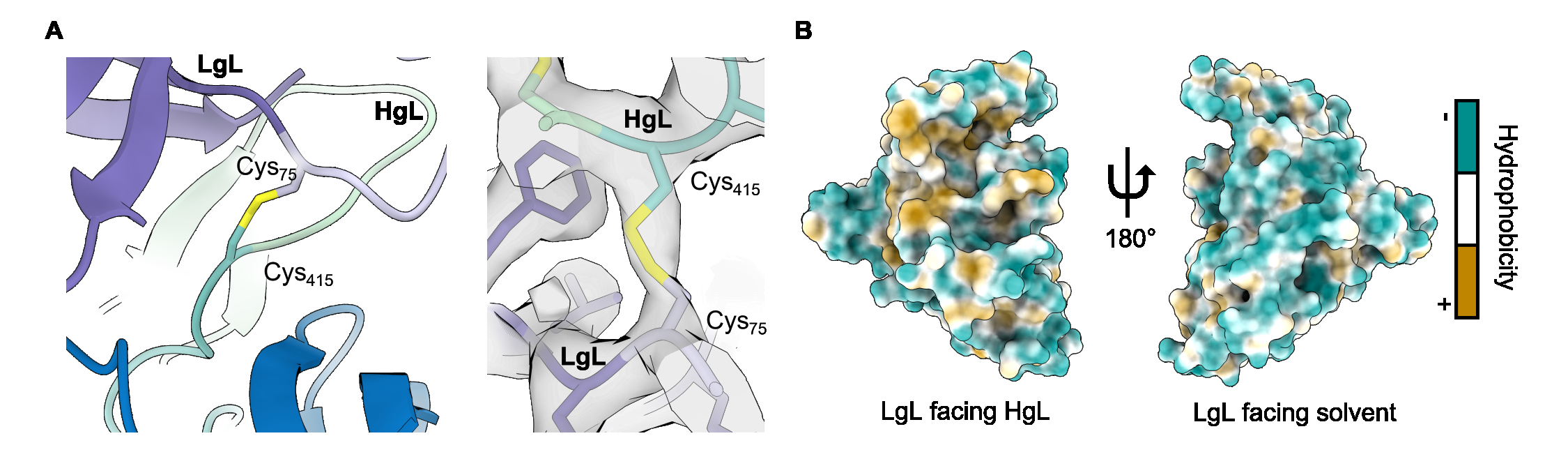

Supplement: S5 Fig — (A) The single intramolecular disulphide bond links HgL and LgL. (B) Surface hydrophobicity of LgL, showing the interface between LgL and HgL (left) and solvent exposed side of LgL (right). (TIF) [file ppat.1013948.s005.tif]

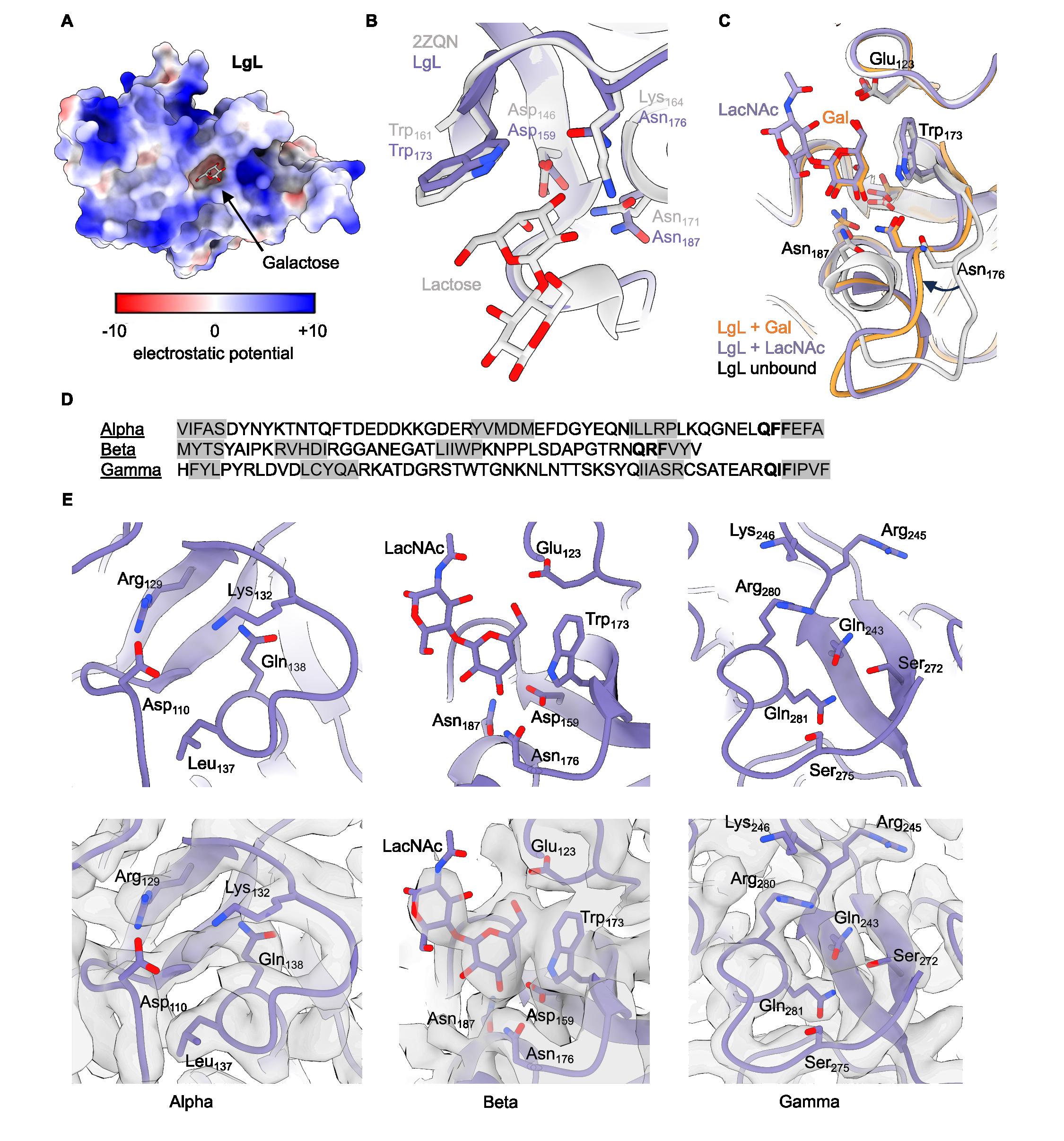

Supplement: S6 Fig — (A) Surface electrostatic representation of galactose-bound LgL. (B) Cartoon representation of the alignment between the structural models derived from the cryo-EM reconstruction of carbohydrate-bound HgL-LgL heterodimer (purple) and a lactose-bound beta trefoil (light grey, PDB code 2ZQN) showing an analogous carbohydrate binding pocket. (C) Cartoon representation of the alignment between the structural models derived from the cryo-EM reconstruction of HgL-LgL heterodimer bound to Gal (orange) and LacNAc (purple), and in the absence of carbohydrate (light grey). (D) Sequence of the three pseudo-symmetric portions of LgL β-trefoil. Residues forming the twelve β-strands of the β-trefoil are highlighted in grey. The QXF motif is displayed in bold. (E) Close-ups on the cartoon representations of the three potential carbohydrate binding pockets of LgL β-trefoil and their overlay with the corresponding cryo-EM density. (TIF) [file ppat.1013948.s006.tif]

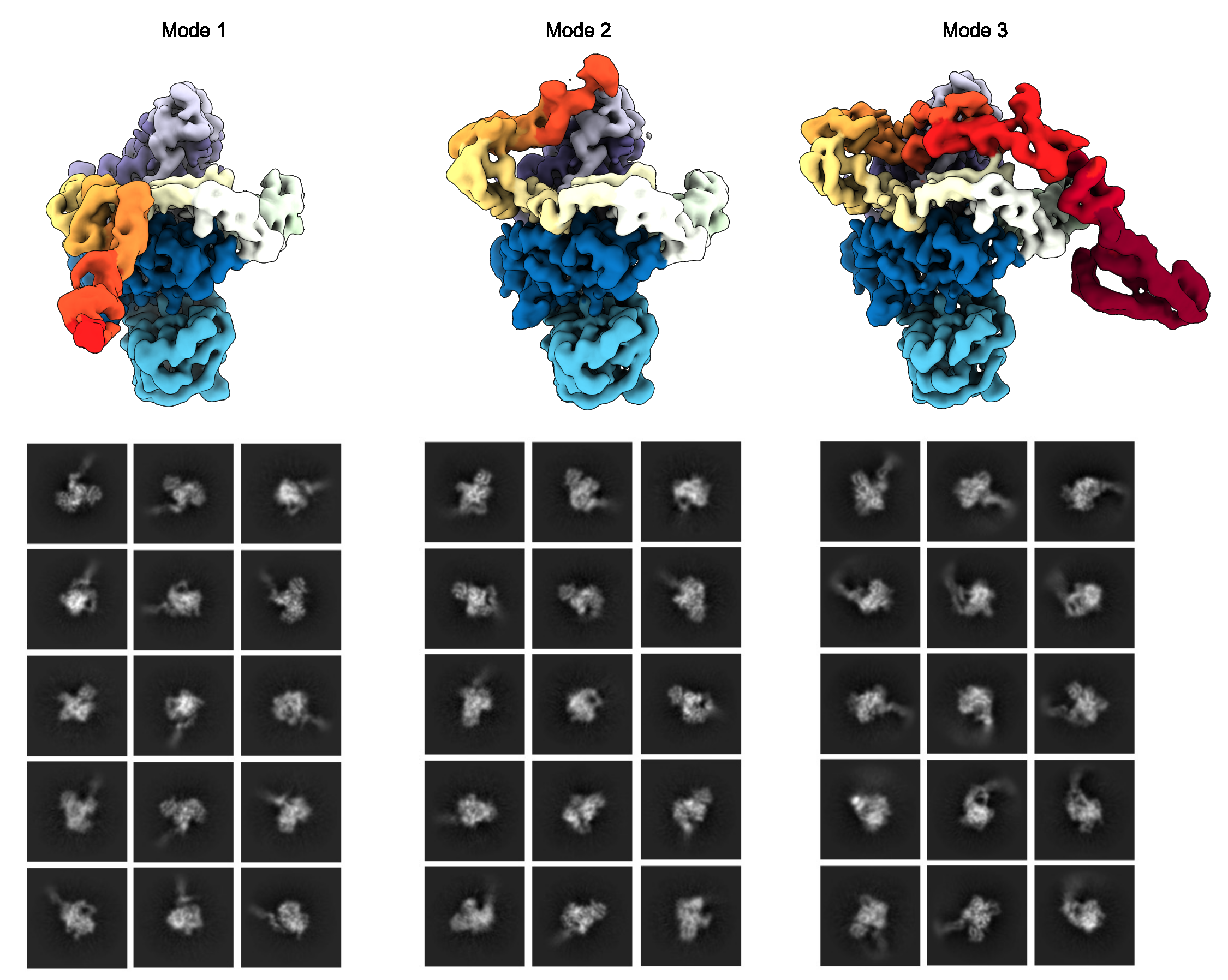

Supplement: S7 Fig — (TIF) [file ppat.1013948.s007.tif]

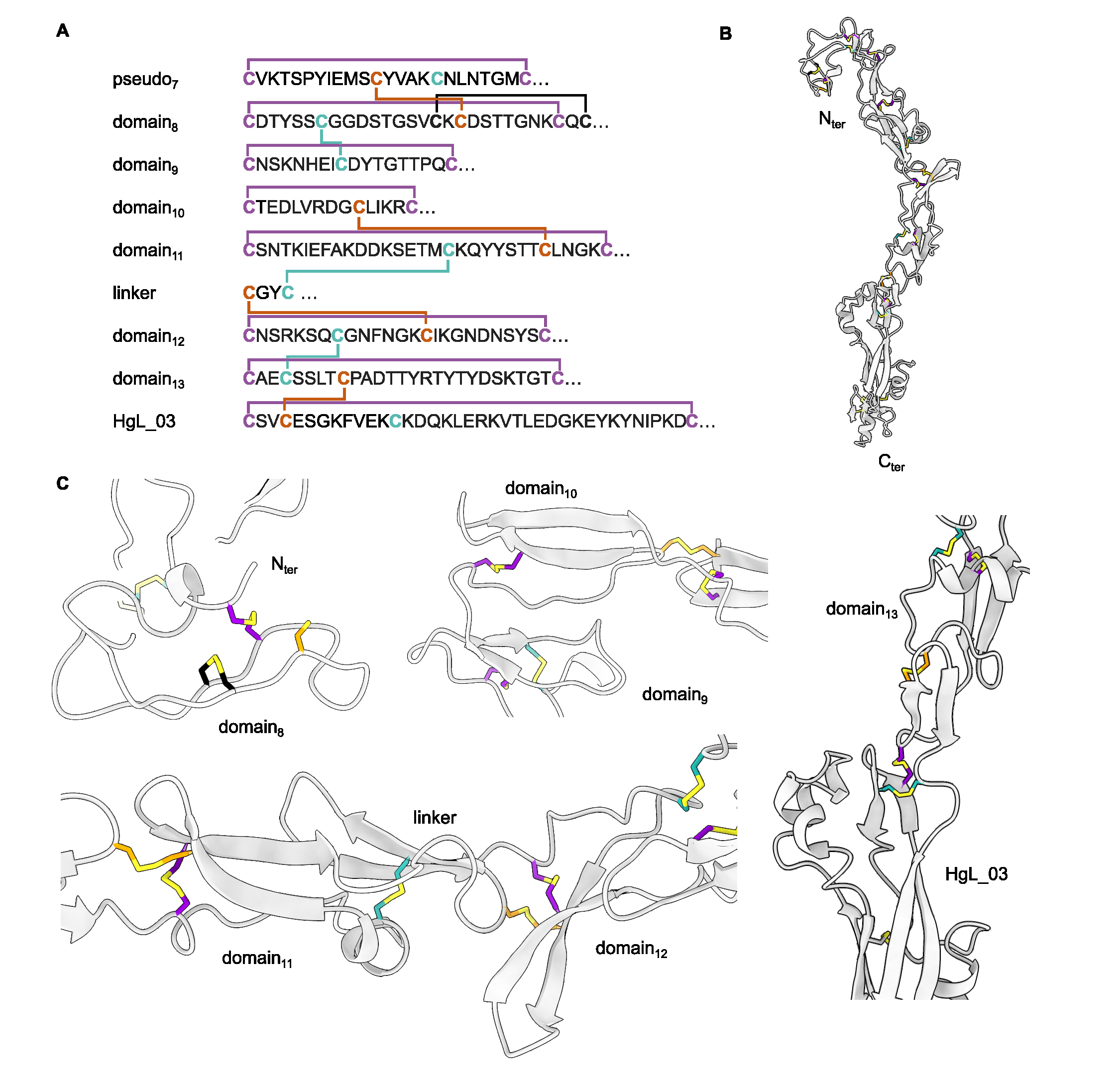

Supplement: S8 Fig — (A) Sequence of each domain of the arm and the N-terminally adjacent pseudo-domain of the arc, with coloured cysteines. Bridges between cysteines represent disulphide bonds. (B) Cartoon representation of the resolved domains of HgL arm with disulphide bonds coloured as (A). (C) Close-ups on the different domains and their interfaces. (TIF) [file ppat.1013948.s008.tif]
