# Supplementary material for: Structural basis for carbohydrate recognition by the Gal/GalNAc lectin of Entamoeba histolytica involved in host cell adhesion
Source: PLoS Pathog. 2026 Feb 24;22(2):e1013948. doi: 10.1371/journal.ppat.1013948 (PMC12948311; doi:10.1371/journal.ppat.1013948)

***S1 Table. Crystallographic data collection and refinement statistics for HgL_03 alone and co-crystallized with Gal, GalNAc or LacNAc***


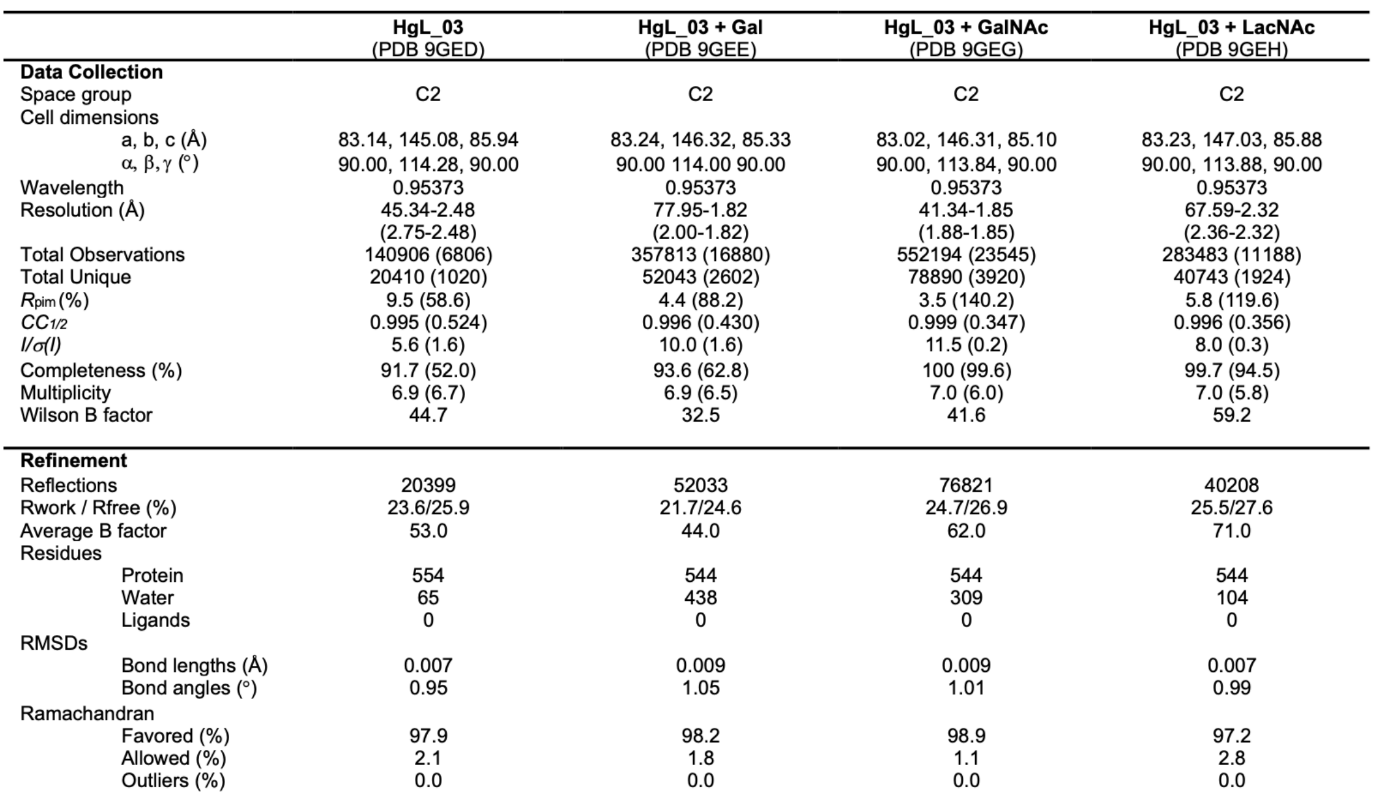

Supplement: S1 Table — (DOCX) [file ppat.1013948.s009.docx]
