# Supplementary material for: Structural basis for carbohydrate recognition by the Gal/GalNAc lectin of Entamoeba histolytica involved in host cell adhesion
Source: PLoS Pathog. 2026 Feb 24;22(2):e1013948. doi: 10.1371/journal.ppat.1013948 (PMC12948311; doi:10.1371/journal.ppat.1013948)
Supplement: S2 Table — (DOCX) [file ppat.1013948.s010.docx]

***S2 Table. Cryo-EM data collection, refinement, and validation statistics for the Gal/GalNAc lectin***

***
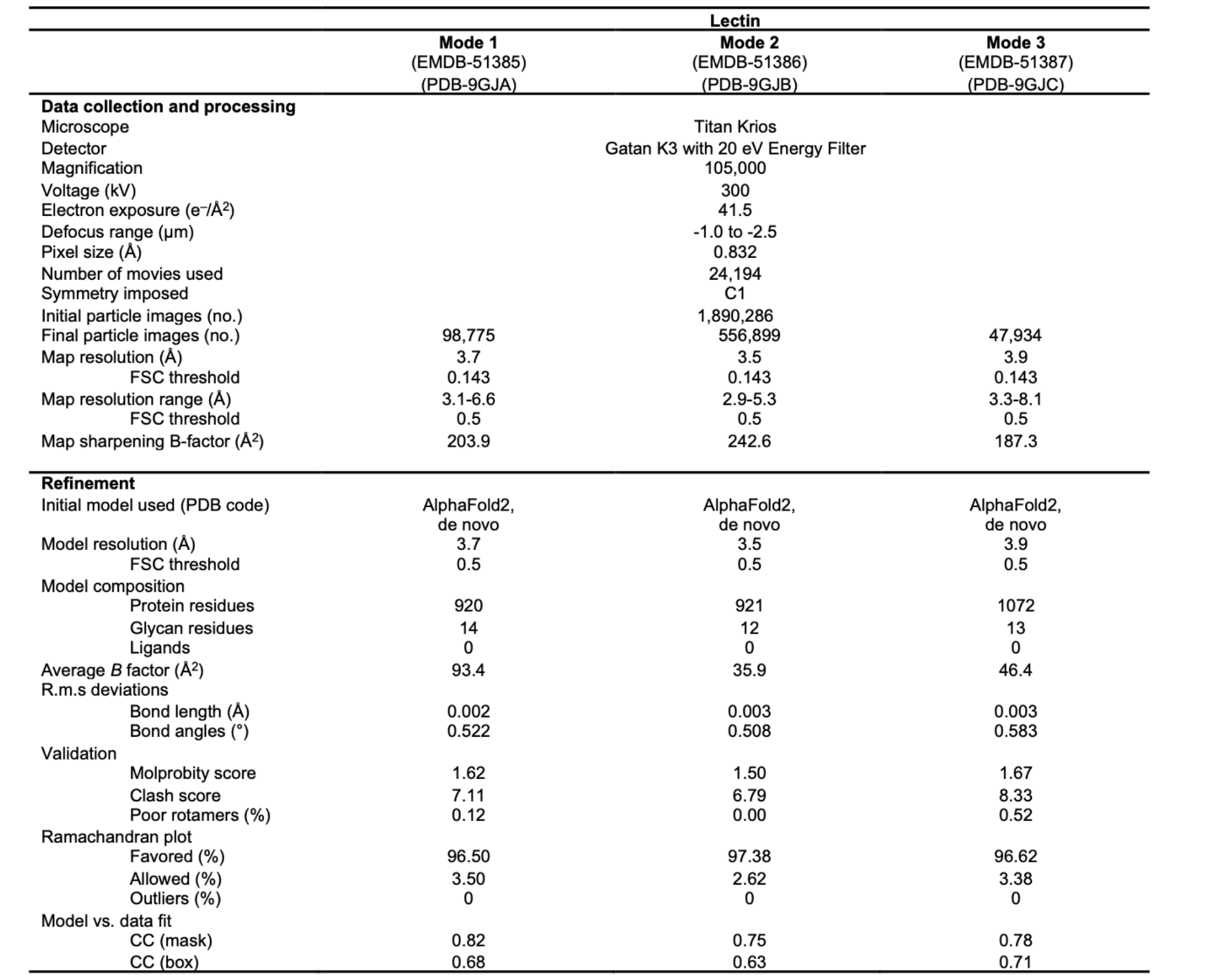
***
