# Supplementary material for: Structural basis for carbohydrate recognition by the Gal/GalNAc lectin of Entamoeba histolytica involved in host cell adhesion
Source: PLoS Pathog. 2026 Feb 24;22(2):e1013948. doi: 10.1371/journal.ppat.1013948 (PMC12948311; doi:10.1371/journal.ppat.1013948)
Supplement: Attachment — Submitted filename: response to reviewers.docx [file ppat.1013948.s014.docx]

**Reviewers' Comments:**

Reviewer #1: The work primarily focuses on structural characterization of the extracellular part of a heterodimeric complex of a membrane protein that plays a crucial role in the host interaction of the parasite Entamoeba histolytica. This structure is noteworthy in several aspects. Firstly, a novel structural fold is identified, which is rare enough to be highlighted. Secondly, this work identifies an error in the allocation of a carbohydrate binding site. It was previously believed to be on the larger subunit, but it is actually on the smaller one. The authors present strong evidence for this and identify the new site with the ligand inside. Finally, they have identified an elongated arm with multiple potential conformations that could play a significant role in the accessibility of this carbohydrate binding motif, thereby facilitating the interaction with the host and the initiation of the parasite invasion.

The strength of this work lies in its meticulous and precise structural characterization, achieved through both X-ray, STD NMR and cryoEM approaches. However, the weakness is the limited functional characterization, primarily limited to structural descriptions. The reader may feel a bit frustrated after the detailed and informative descriptions of these structures, without seeing physiologic characterization that aligns with the proposed hypothesis.

However, the work is really well conducted, and these new structures and characterizations are without doubt of great importance in the corresponding field and studies around the invasive mechanisms of this parasite. I have no reason not to recommend the publication of this nice work.

We thank the reviewer for their positive review of our manuscript and for highlighting the ‘strong evidence’ for our discovery of the sugar binding site on the lectin and for pointing out that the work is ‘really well conducted.’

We empathise with the wish that further physiological characterisation had been possible. We wish so too. However, there were two substantial technical challenges which prevented this. The first was that this work had to be conducted using native protein, extracted from *Entamoeba histolytica* cells, as we were unable recombinantly express the heterodimeric lectin. This made the *in vitro* testing of mutants impossible. Secondly, the lectin is encoded by multiple genes for multiple isoforms in the same genome which, together with field-wide limitations in the generation of transgenic *Entamoeba histolytica* meant that we could not generate parasites for functional studies in which the lectin(s) had been mutated. This has limited us to the current structural and *in vitro* study.

Nevertheless, we are grateful for the reviewer’s view that the work should be published and are of the view that the current study forms a complete piece of work, ready for publication.

Reviewer #2: The results reported in this manuscript provide evidence that the sugar-binding site in the Entamoeba histolytica adhesin is located in a beta-trefoil domain in the light chain of the adhesin. The structures reported also provide evidence that the overall structure of the adhesin exists in multiple conformations.

Although the findings contradict previous studies suggesting that the sugar-binding site is located in the heavy chain, the combined EM structures and TrNOE NMR data provide compelling evidence that the binding site is located in the light chain. The extensive structural analysis using both crystallography and EM also provides an overview of the organization of the adhesin. Both of these results represent significant achievements and are generally well supported by the data.

We are grateful to the reviewer for their positive assessment of the work and their assessment that we provide compelling evidence for our discovery and that the manuscript presents significant achievements.

Reviewer #3: The manuscript reported the first crystal structure of the carbohydrate binding binding domain (CRD) of HGL (HgL_03) from Gal-GalNAc cell surface lectin, from the enteric parasite Entamoeba histolytica and cryoelectron microscopy structures for the native lectin (HGL-LGL heterodimer) extracted from the amoeba. The authors also solved the structures of the co-crystals grown in the presence of Gal/GalNAc or LacNAc.

The structure of the lectin consisted of a core ordered part made up of N terminal half of HGL and LGL bound to it. HGL in the hetero-dimer revealed existence of a bacteiral lipoprotein like domain in the N-terminal ordered core followed by a Von Willebrand-like domain. Further C terminal Cysteine-rich region consists of a hinge and followed by a dynamic arm. The arm was shown to adopt three distinct conformations, mode 1-3. LGL showed beta-trefoil fold and was found to be covalently linked to HGL via disulphide bond.

The lectin is known for its very crucial role in adhesion of the amoeba to the host gut via its carbohydrate binding function and thereby promoting amoebic pahthogenesis. The author studied carbohydrate binding by HgL_03 and showed that it does not show any binding to Galactose or LacNAc. Instead, LgL showed binding to Galactose. Based on the cryo electron microscopy electron density map, the authors modeled Gal and LacNAc binding to LGL.
The results reported in the manuscript certainly leads to an advancement in terms of structural information about one of the most well studied amoebic antigens. Interestingly, it reported some observations which is in sharp contrast to the existing literature. It also revealed the existence of domains with implications in lipid transport possible hinting new functions of the lectin.

However the manuscript may gain more strength with more supporting observations using alternative experimental approach as well as inclusion of some of the results as controls.
The method section may need more attention with elaboration of the text and inclusion of results as supplementary information.

We thank for the reviewer for their positive assessment of the work. While we too wish that additional experimental approaches had been possible, as outlined in the response to reviewer 1, technical challenges have not allowed this.

**Part II – Major Issues: Key Experiments Required for Acceptance**
Please use this section to detail the key new experiments or modifications of existing experiments that should be absolutely required to validate study conclusions.

Generally, there should be no more than 3 such required experiments or major modifications for a "Major Revision" recommendation. If more than 3 experiments are necessary to validate the study conclusions, then you are encouraged to recommend "Reject".

Reviewer #1: nothing to ask for

Reviewer #2: (1) The manuscript is focussed on the experimental analysis and does not provide major biological insights because of a lack of links between the structural features observed and the biological functions of the adhesin. Thus, the manuscript does not present the work in a very accessible or compelling way and needs significant refocus, as discussed in the next section.

We think that the reviewer here is referring to the minor comments section and confirm that we have addressed their comments below.

(2) Analysis in vitro and in vivo of the effects of mutations in the sugar-binding site and the proposed hinge region would substantiate the conclusions and provide at least some linkage to biological functions.

Sadly, for technical reasons, the experiments suggested by the reviewer are not possible.

We made extensive attempts to express recombinant heterodimer, testing all the usual bacterial and eukaryotic expression systems, including HEK293 and S2 cells. We also tested a range of tagging systems, including use of tags which drive heterodimerisation. We were able to express heavy chain constructs which, when studied through 2D class averages, resembled native heterodimer purified from *Entamoeba histolytica*. However, we could not express the light chain or any recombinant heterodimer. We have extensive experience at expressing challenging parasite surface protein complexes and this is a particularly difficult one! Indeed, the structure shows why the light chain cannot be expressed alone, as it is intimately associated with the heavy chain. As the sugar binding site is in the light chain, this prevented us from mutating the site in recombinant protein to test our model.

The other option is to mutate the lectin in *Entamoeba histolytica* cells. However this was also not possible, firstly due to the poorly developed transgenics of this organism and secondly because the light chain is encoded by multiple genes for different isoforms, as a gene family rather than a single gene.

Therefore, sadly, we cannot provide the experimental data which the reviewer suggests. However, we agree with the reviewers that we have ‘strong evidence’ and ‘compelling evidence’ for our conclusions through high quality structural data and therefore are not concerned that the technical limitations on mutation undermine the value of the work.

Reviewer #3: 1. Authors could use Gal-GalNAc instead of LacNAc in all carbohydrate binding experiments, as this di-saccharide showed the most effective binding to the CRD as compared to Gal or GalNAc in the earlier studies, reflected from multiple experimental approaches including cell adhesion/agglutination experiments, ITC,etc.

2. A concentration dependent binding experiment could be performed to determine affinity for the carbohydrates by the native hetero-dimer.

The reviewer proposes a couple of experiments here to probe the affinity and specificity of the lectin. These experiments are plausible, using our HDX-NMR assay, but would be very technically intensive as these experiments can only be conducted using lectin produced from *Entamoeba histolytica* cells. The amount of material that would be required to generate full binding curves to measure affinities is very large relative to what we have already generated and, while these experiments would produce data, they would not change the conclusions of our paper, which is not about the carbohydrate affinity of the lectin.

In addition, the reviewer suggests that we repeat our analysis using Gal-GalNAc as well as LacNAc as he has previously studied the binding of Gal-GalNAc to the lectin using ITC. We chose to conduct the work using LacNAc as this has previously been demonstrated to be a ligand for the lectin. Early studies (i.e. Adler et al 1995 PMID 7890626) showed that LacNAc is highly effective at preventing hemagglutination and (i.e. Li et al 1989 PMID 2535835) inhibiting trophozoite adherence to CHO cells and cytolysis of wild-type cells. Indeed, LacNAc is also the terminal glycan of the mucins, which are the native target of the lectin. It is therefore our view that the disaccharide studied in our work is physiologically valid and there is no transformative value to our work to repeat with Gal-GalNAc or other sugars. Indeed, our work is not about glycan specificity of the lectin.

Our view is therefore that additional experiments to measure affinities for different disaccharides are outside the aims of this paper.

**Part III – Minor Issues: Editorial and Data Presentation Modifications**
Please use this section for editorial suggestions as well as relatively minor modifications of existing data that would enhance clarity.

Reviewer #1:

1) Identify N and C-terminal motifs of the protein in Figure 1.

We have now added the N and C termini to Figure 1B.

2) The authors mention in the text the existence of 3 QXF sequence motifs in Lgl which traditionally mediated binding to Gal/GalNAc containing sugars. However, they do not pinpoint those in Figure 4 where the carbohydrate binding mode is presented. It creates some confusion and needs to be clarified in one way or another on the real role of these motifs in the particular case of this protein.

We have now updated the Figures and show the locations of the QxD sites in another β-trefoil lectin from Entamoeba histolytica (6ifa) for comparison. This is now in Figure 2E. The QxD motifs are representative of the β-trefoil fold.

We do show a comparison of the light chain with one β-trefoil lectin bound to sugar (Figure S6BC) but are not of the view that we need to show a panel of such Figures.

Reviewer #2: (1) Neither the title or abstract highlight key findings for potential readers. For instance, the title could refer to sugar-binding "adhesin" to reflect its biological significance and the abstract makes no mention that the sugar-binding domain falls into a known class of protein folds that support this activity.

We have increased the abstract by adding ‘involved in host cell adhesion’. We have also added a couple of clauses to the abstract to point out that the light chain “adopts a β-trefoil fold found in other lectins” and that the arm ‘may be modulated by sugar binding’. We are confident that the title and abstract are appropriate.

(2) No view of the overall organization with respect to the membrane is shown and it is not indicated if the two subunits are encoded in separate genes or are produced by proteolysis.

We have added ‘each expressed from a different gene’ to line 29-30.

We have added ‘The transmembrane helix lies at the end of this arm and is not visualised’ to lines 196-7. As the transmembrane attachment is not visible, we cannot add it to structure figures. However, we have highlighted its location in the schematic on Figures 1A, 2A and 4A.

(3) A great deal of the manuscript is given over to descriptions of negative results. Roughly the first third of the Results section, including the one of the four figures are all negative results before anything positive is reported.

We do not agree that the first part of the manuscript is ‘negative’. It presents the first structure of one of the most intensively studied parts of the lectin, the CRD. It also presents the discovery that, while we can detect sugar binding to the native lectin, we do not observe sugar binding to the previously proposed carbohydrate binding domain. This is essential data which provides the foundations for our subsequent demonstration that the sugar binding site is in the light chain and we remain of the view that this is most effective way in which we can present our findings to help the reader to see the discovery.

(4) In addition to over-emphasizing the negative results, the distribution of information between the main text and the supplement make the main text very hard to follow without reference to the supplement. Several of the supplemental figures would be better in the main text. For example, the definition of the expressed regions in Supplementary Figure 1 is critical for understanding results shown in the main text figures. The supplementary figures often make the same points as the main text, but illustrate them better. In addition to Supplementary Figures 1, Supplementary Figures 5-9 would all be better in the main text.

We have brought some of the supplementary material into the main paper, including the content of Figure S1 (now in Figure 1) and Figure S6A and B (now In Figure 2). We have also rearranged the manuscript such that we describe the sugar binding site before we present the arm conformations as this gives a better flow after moving data between figures.

Our view is that Supplementary Figures 4 and 7-9 are in the right location in supplementary information. These will be of interest to a specialist reader but would disrupt the flow for a less specialist reader.

(5) In the Results section, a great deal of space is given over to descriptions of the topology, which can be discerned from the figures, rather than information about what model was used for molecular replacement: the fact that an alpha-Fold model was used merits mention here rather than just in the Methods section.

Our view is that the description of how we used molecular replacement is best in the methods section of the paper, where it is available for a specialist reader.

(6) The Discussion re-summarizes results, including the negative ones, but does not provide new insights. Only the final sentences of the third and fourth paragraphs present implications of the results for future work.

The discussion correctly describes what we consider to be the outcomes of our study, as is the prerogative of an author.

(7) There are many missed opportunities for insights, such as what was the previous evidence for an alternative binding site, comparison with another galactose-binding beta-trefoil lectin E. histolytica lectin [reference 26], the unusual and intriguing role of the glycan at a domain interface, and the accessibility of the sugar-binding site for interaction with large mucin molecules in the context of the whole protein, the membrane, and the flexible arm.

We reviewed our description of comparisons to the lectin described in reference 26 and have made a few changes. In lines 149-154, we had described the similarity and differences in structure in the original version of the manuscript. However, we had omitted to clearly state that this protein is a lectin. We have now fixed this in line 151.

Later in the manuscript, we had already compared the light chain to other β-trefoil containing lectins (lines 172-187) and our view is that this is a suitable level of discussion.

We have now altered Figure 2E to show a comparison of the light chain with another β-trefoil containing lectin from *Entamoeba histolytica*.

In our view this is suitable level of comparison of our findings with previously identified structures, which credits the work of others while focusing on our own discoveries.

(8) There are some significant misstatements and the writing is repetitive, with roughly ten sentences beginning "Indeed".

We disagree and are of the view that the writing is accurate and of high quality.

The title is somewhat misleading in referring to "the" Gal/GalNAc-binding lectin, given that there is at least one other such lectin as noted above.

We have added ‘involved in host cell adhesion’ to the title to help, as the reviewer suggested earlier.

The statement on page 5 that the beta-trefoil is preceded by a region "without secondary structure" suggests a disordered region, while in fact a region without regular secondary structure elements is meant.

We disagree that ‘without secondary structure’ suggests a disordered region. It suggests that the polypeptide is not part of a secondary structural element such as a helix or sheet.

The term "structural homology" is used in several places where structural similarity is meant.

We have changed this in lines 72 and 222.

The significance of some details provided in the Methods section, such as the fact that NMR tubes were "placed in 96-tube racks", is unclear.

The NMR machine has 96-tube racks to allow automatic analysis of multiple samples. We have removed this clause as it is unimportant.

On page 11, PDB entry 6IFB is cited as a model used for positioning galactose in the binding site, but this entry contains rhamnose, not galactose.

The reviewer is correct and 6IFB was not used to help position galactose. We have corrected in the methods (line 439). 5Y97, 4IZX, 3NBD, 2ZQN and 1PUU were all used to guide this docking, providing more than enough information.

The legend to Figure 3 refers to a Panel E that does not seem to exist.

We have removed this panel and checked all other figure legends.

Reviewer #3: All the points stated below could be included in the supplementary materials or discussion/results as appropriate.

1. Elaborate all the methods with inclusion of data from controls, protein purification/extraction, protein extraction/purification PAGE/Western blot images. Provide the details of Purification reagents, detergents etc/buffers for extraction of native hetero-dimer.

We are confused about this comment as these details of the purification are already outlined in the methods section (i.e. lines 277-301). We are confident that the reviewer will find all they need there.

We are also confused about exactly what data ‘from controls, protein purification/extraction, protein extraction/purification PAGE/Western blot images’ the author suggests is missing. We have included the data in the manuscript which justifies the conclusions. We have not included a purification gel but include it here for the reviewer to judge. As this work presents the cryoEM structure, we think that the gel is redundant.

2. Show detailed binding sites for LacNAc on LGL. Discuss if data suggests that LacNAc and Gal-GalNAc will have similar affinity based on the structure. Do HGL atoms interact with the carbohydrate atoms?

At the resolution obtained by cryoEM, we are confident about the location of the sugar binding site but to propose a precise model for binding would be to over-interpret the structural data. We therefore prefer not to do this. However, we can confirm that HGL does not interact with the disaccharide.

3. Provide coulomb density map for the LacNAc binding site including the binding site residues which are within 6A (0.6nm) from any of the LacNAc atoms. Provide the same without modelling LacNAc into the density. At what sigma level the coulomb data was used to model the carbohydrate?

The Coulomb densities were already shown in Figure 4, now in Figure 3D, clearly showing the density for the disaccharide or monosaccharide in pink-coloured density. We have now added the sigma level for the coulomb density in the legend to this figure.

4. Provide NMR raw data (1D H NMR) highlighting the Amino (backbone) and Methyl (Core) shifts to provide support for your statement that the extracted/purified proteins are properly folded.

We did not include this data in the manuscript as our view is that it is redundant as we have crystal structures or cryoEM structures of the key proteins tested, but we include it here.

5. Discuss if the symmetry related molecules in the crystal structure of HGL_03 has impact on carbohydrate binding.

We cannot be entirely sure that crystal packing does not interfere with sugar binding to HGL_03. However, it is noteworthy that we co-crystallised with sugar rather than soaking (lines 80-82) and so it is not crystal packing which prevents sugar from accessing its site. Instead, crystals formed in the presence of high concentrations of sugars, which would not happen if the sugar tightly bound in such a way as to block a crystal contact. In addition, we did not overinterpret this data as we simply state that we were not ‘able to detect the presence of any ligand). We then conducted STD-NMR and observed no sugar binding while binding was observed to the native heterodimer. These findings, using multiple independent methods, give the same result, suggesting that the lack of sugar density in the light chain is not due to crystallisation artifacts.

6. Authors have used Cys-Ser mutant for Hgl_03, discuss if this could impair loss in carbohydrate binding, given the fact that earlier CRD characterization studies (referred in this manuscript) used wild type Cys containing proteins. If oligomerization of HGL_03 is required to constitute the carbohydrate binding site, this may be feasible.

In the design of this construct, we mutated a cysteine (972) which is predicted by AlphaFold to disulphide bond to a cysteine absent in the CRD construct (1029). We have clarified this in lines 65-6. Therefore, the C972S mutation removes the potential for problematic and non-physiological disulphide bonding which would not occur in native lectin. We therefore are confident that we made the right decision. Whether the presence of this cysteine in previous studies affected the folding of the CRD and the outcome of experiments is not something which we can comment on as we have not tested this.

7. Do the cryo-electron microscopic data account for the trans-membrane and cytosolic part of the complex? elaborate.

We have now added in lines 196-7 that we do not observe the transmembrane helix.

8. The long standing experimental outcomes in the amoeba field depicts HGL to harbour the CRD, however the current study instead pointing towards LGL, could there be possibility of both hoarbouring the sites with very dissimilar in vitro affinities but still physiologically relevant in their capacity to carbohydrate binding in the host gut during amoebic infection.

As outlined in our manuscript, we see no evidence for this hypothesis. Our evidence is laid out in the discussion (lines 219-228). However, we are careful to write ‘our data cast doubt…’ rather than stating that it disproves there being a sugar binding site in the heavy chain. We prefer not to speculate further in the manuscript, but rather to present our clear data and its interpretation.
